# Extreme Heat Exposure and Adolescent Cognitive Function

**DOI:** 10.31586/ojn.2025.1247

**Published:** 2025-01-16

**Authors:** Shervin Assari, Hossein Zare

**Affiliations:** 1 Department of Internal Medicine, Charles R. Drew University of Medicine and Science, Los Angeles, CA, United States; 2 Department of Family Medicine, Charles R. Drew University of Medicine and Science, Los Angeles, CA, United States; 3 Department of Urban Public Health, Charles R. Drew University of Medicine and Science, Los Angeles, CA, United States; 4 Marginalization-Related Diminished Returns (MDRs) Center, Los Angeles, CA, United States; 5 Department of Health Policy and Management, Johns Hopkins Bloomberg School of Public Health, Baltimore, MD, United States; 6 School of Business, University of Maryland Global Campus (UMGC), Adelphi, MD, United States

**Keywords:** Climate Change, Extreme Heat Exposure, Cognitive Function, Child Development, Socioeconomic Status (SES), Neighborhood SES, Racial Disparities, Environmental Stressors, Climate Change, Cumulative Risk

## Abstract

**Background:**

Extreme heat exposure is an increasing public health concern, particularly in the context of climate change. Limited research has explored its impact on children’s cognitive outcomes. This study investigates the association between extreme heat exposure and cognitive function in 9–10-year-old children, using data from the Adolescent Brain Cognitive Development (ABCD) study. Additionally, we assess whether this effect is independent of socio-demographic factors such as race, family socioeconomic status (SES), and neighborhood SES.

**Methods:**

Data were drawn from the ABCD study, comprising over 10,000 children aged 9–10 years. Cognitive function was assessed through standardized cognitive tests, while extreme heat exposure was estimated using geographic and climate data. Structural equation modeling (SEM) was employed to examine the direct effects of heat exposure on cognitive outcomes and to account for potential confounding variables, including race, family SES, and neighborhood SES.

**Results:**

Black families, low SES households, and children from low SES neighborhoods were disproportionately exposed to extreme heat. Extreme heat exposure was significantly associated with lower cognitive function in children, and this association remained robust even after adjusting for socio-demographic factors.

**Conclusions:**

Extreme heat exposure is linked to diminished cognitive function in children, particularly among socio-economically disadvantaged and marginalized populations. Given the increasing frequency of extreme heat events due to climate change, future research should further explore these implications for children’s cognitive outcomes. Policy interventions that improve access to cooling infrastructure, expand green spaces, and prioritize at-risk populations are critical to mitigating the adverse cognitive effects of extreme heat in low SES communities.

## 1. Introduction

Climatic change is a public health challenge. Since the 1850s, temperatures have surged to their highest levels globally. Forecasts by the Intergovernmental Panel on Climate Change (IPCC) suggest that the frequency and intensity of extreme temperatures will continue to rise, driven largely by climate change [1]. The economic consequences of extreme weather events include unexpected operational costs for urgent adaptations, increased firm costs, reduced local demand, diminished agricultural yields, lower labor productivity, increased absenteeism, and decreased local spending [2–5]. Global warming in the past decades has particularly heightened concerns about a wide-range of economic, health and environmental impacts of extreme weather, for low-income and Southern areas [6]. In these resource- constrained areas, humans can minimally adapt and cope with such environmental challenges.

Exposure to extreme heat is not rare. It is estimated that half of the global population which includes more than 1 billion workers, are exposed to extreme heat episodes, and a third of this population are experiencing negative health effects [6]. The health ramification of extreme heat exposure is also alarming [7, 8]. Exposure to extreme heat is associated with elevated morbidity and elevated mortality. It also adversely affects pregnancy outcomes as well as mental health. Exposure to extreme heat and associated heat stress can reduce physical work capacity and motor-cognitive performance in individuals across age groups. Exposure to extreme heat reduces workers’ productivity and increases the risk of occupational accidents [6].

Health risks of extreme heat exposure are preventable through appropriate heat action plans that incorporate economic as well as biophysical solutions and strategies [6]. Each year, extreme heat events cause numerous excess deaths, particularly during the summer season, worldwide. Unfortunately, due to the ongoing climate change, these trends are projected to worsen over the next decades. In tropical regions, warming has pushed physiological limits related to heat tolerance which itself poses additional survival risks in the coming decades [6]. The challenges due to climate change is related to other global trends such as population growth, aging, urbanization, and socioeconomic development. Urban populations are particularly at risk, facing increased temperatures due to anthropogenic heat, vehicular transport, and heat waste from buildings [6].

According to the Centers for Disease Control and Prevention (CDC), children and adolescents are particularly vulnerable to the adverse effects of exposure to extreme heat. According to one study, high maximum temperatures (Tmax) increase the risk of emergency department visits. To be more specific, the study reported an excess risk of 2.6% (95% CI: 2.2-3.0) for emergency visits for each 13°F increase in Tmax. These effects are particularly strong for 1-4-year-olds. Increased emergency utilization may be due to heat-specific diagnoses (16.6% [95% CI: 3.0-31.9]), general symptoms (10.1% [95% CI: 8.2-11.9]), infectious diseases (4.9% [95% CI: 3.9-5.9]), and injuries (5.1% [95% CI: 3.8-6.4]), which seem to be similar across diverse race/ethnicity subgroups [9, 10].

Children and adolescents exposed to extreme heat may experience disrupted children’s activities, such as play and socialization, leading to reduced community presence and altered social relationships. These effects are more pronounced for children living in poverty, who may lack access to mitigating resources like air conditioning. Despite these significant impacts, the literature on the influence of climate change and extreme heat exposure on youth development remains sparse. According to a 2014 systematic review [10], some of the health effects of extreme heat exposure are not well understood. While pediatric conditions such as renal and respiratory diseases, electrolyte imbalances, and fever are known effects of exposure to heat wave, such effects on cognitive, behavior, and emotion of children are still unknown. We need to study factors such as age, sex, socioeconomic, or family background that may influence children’s vulnerability to heat waves and their adaptation or mitigation strategies [11].

As climate change continues to increase the occurrence and undesired consequences of extreme heat exposure across the globe [12–15], we need studies that help us better understand the impact of such heat exposure on child development, in terms of cognitive function, behaviors, and emotions.

In this study, we aimed to fill this research gap by examining effects of extreme heat exposure on children’s outcomes. For this study, we leveraged economically and race and ethnically diverse data from the Adolescent Brain Cognitive Development (ABCD) [16–25] study. We have previously shown that neighborhood socioeconomic status (SES) [26, 27], financial difficulty [27], peer deviance [28], and puberty [29] may act as mediators for the effects of extreme heat exposure on youth delinquency.

## 2. Methods

### 2.1. Design and Sample

We conducted a secondary analysis using data from the Adolescent Brain Cognitive Development (ABCD) study [16–25], a national longitudinal study of a racially and economically diverse cohort of pre-adolescent children. The ABCD study’s methodology has been thoroughly documented elsewhere. Advantages of the ABCD dataset include its longitudinal design, national scope, large and diverse samples in terms of race, SES, and geographic distribution. Participants were primarily recruited from schools.

### 2.2. Analytical Sample

The analytical sample consisted of participants across race, ethnic, and SES groups. Participants were 9–10-year-old at baseline. 11,878 children entered our analysis.

### 2.3. Ethics

The study was approved by the Institutional Review Board (IRB) of the University of California, San Diego (UCSD). Assent was obtained from all participating adolescents, and informed consent was obtained from their parents.

### 2.4. Study Variables

The study variables included race, demographic and socioeconomic factors, adversities, and substance use.

#### Race:

Parents reported the race and ethnicity of their children. This was a categorical variable with White as the reference category.

#### Age:

Age at baseline was calculated in years. It was based on the number of years between birth and study date.

#### Sex:

This was a dichotomous variable with 1 for male and 0 for female.

#### Socioeconomic Status:

Socioeconomic status was defined a primary component analysis using the following variables. This variable was then dichotomized (0 for high and 1 for low SES).

#### Parental Educational Attainment:

Participants were asked, “What is the highest grade or level of school you have completed or the highest degree you have received?” Responses were 0 = Never attended/Kindergarten only; 1 = 1st grade; 2 = 2nd grade; 3 = 3rd grade; 4 = 4th grade 4; 5 = 5th grade; 6 = 6th grade 6; 7 = 7th grade 7; 8 = 8th grade; 9 = 9th grade; 10 = 10th grade 10; 11 = 11th grade; 12 = 12th grade; 13 = high school graduate; 14 = GED or equivalent diploma; 15 = some college; 16 = associate degree: occupational; 17 = associate degree: academic program; 18 = bachelor’s degree (ex. BA; 19 = master’s degree (ex. MA; 20 = professional school degree (ex. MD; 21 = doctoral degree. This variable was an interval measure with a range between 1 and 21, with a higher score indicating higher educational attainment.

#### Family Income:

Family income was a continuous measure ranging from 1 to 10, with a higher score indicating higher income. The exact question was, “What is your total combined family income for the past 12 months? This should include income (before taxes and deductions) from all sources, wages, rent from properties, social security, disability and veteran’s benefits, unemployment benefits, workman”. Responses included 1 = Less than $5000; 2 = $5000; 3 = $12,000; 4 = $16,000; 5 = $25,000; 6 = $35,000; 7 = $50,000; 8 = $75,000; 9 = $100,000; 10 = $200,000.

#### Financial Stress:

Financial difficulties were assessed through financial difficulties experienced in the past 12 months. Items included inability to afford food, telephone service, rent/mortgage, eviction, utility shutoffs, and unmet medical or dental needs. Responses were binary (0 = no, 1 = yes), and a mean score was calculated, with higher scores indicating higher financial stress.

#### Family Structure:

Parents reported the number of parents in the household and their relationship. This was categorized as 0 for not married and 1 for married households.

#### Cognitive Function:

Cognitive function was assessed in person at baseline using the NIH Toolbox. A latent factor representing cognitive function was constructed using the following measures: (1) Total Composite, (2) Reading, (3) Picture Vocabulary, (4) Picture, (5) Pattern, (6) List, (7) Fluid Composition, (8) Flanker, (9) Crystallized, and (10) Card Sort. All domains demonstrated factor loadings above 0.5, supporting the validity of the latent construct. Higher scores on this latent factor indicated better cognitive function.

### 2.6. Data Analysis

Data analysis was conducted using Stata. Univariate analysis involved reporting the mean and standard deviation (SD) of continuous measures. We used Pearson test to estimate bivariate correlations. Structural equation models (SEM) were used for multivariable analysis, with delinquency as the outcome. Predictor was heat wave. Race, SES, age, and gender were confounders. Collinearity between variables was checked and ruled out (all correlations were below .6). Beta, 95% confidence intervals (CI), and p-values were reported.

## 3. Results

Table 1 summarizes the results from the Structural Equation Model (SEM) testing the association between heat exposure and cognitive function in children, adjusting for socio-demographic factors such as age, sex, family socioeconomic status (SES), neighborhood income, and race/ethnicity.

### Heat Exposure and Cognitive Function:

Heat exposure was significantly and negatively associated with cognitive function (β = −0.018, SE = 0.008, 95% CI: −0.033 to −0.003, p = 0.018). This finding indicates that children exposed to extreme heat had slightly lower cognitive function scores, even after adjusting for other factors.

### Family Socioeconomic Status (SES) and Cognitive Function:

Children from low SES households had significantly lower cognitive function (β = −0.163, SE = 0.009, 95% CI: −0.180 to −0.146, p < 0.001), demonstrating a strong negative impact of household SES on cognitive outcomes.

### Neighborhood Income and Cognitive Function:

Higher neighborhood income was positively associated with cognitive function (β = 0.121, SE = 0.008, 95% CI: 0.105 to 0.137, p < 0.001), suggesting that living in more affluent neighborhoods confers cognitive advantages for children.

### Race/Ethnicity and Cognitive Function:

Latino children had lower cognitive function compared to White children (β = −0.066, SE = 0.008, 95% CI: −0.082 to −0.050, p < 0.001). Black children showed the largest cognitive deficit (β = −0.190, SE = 0.009, 95% CI: −0.206 to −0.173, p < 0.001). Asian children outperformed White children in cognitive function (β = 0.034, SE = 0.007, 95% CI: 0.019 to 0.048, p < 0.001).

### Age and Cognitive Function:

Age showed a strong positive association with cognitive function (β = 0.525, SE = 0.004, 95% CI: 0.517 to 0.533, p < 0.001). As expected, older children performed better on cognitive tasks.

### Sex and Cognitive Function:

Male children had significantly lower cognitive function scores compared to female children (β = −0.015, SE = 0.007, 95% CI: −0.029 to −0.001, p = 0.041), indicating a small but significant disadvantage for boys.

### Measurement Models:

All the cognitive measures demonstrated good reliability, with high factor loadings across subdomains. For example, the total composite cognitive score had a factor loading of 0.961, while specific measures such as fluid composition (0.871) and crystallized intelligence (0.886) also showed strong loadings, indicating high measurement accuracy across domains.

These results highlight the detrimental effects of extreme heat exposure on cognitive function, particularly among socioeconomically disadvantaged and minority groups.

## 4. Discussion

This study explored the association between extreme heat exposure and cognitive outcomes among 9–10-year-old children in the Adolescent Brain Cognitive Development (ABCD) study [16–25]. We also tested if this association is independent of socio-demographic characteristics that may impact vulnerability of children and correlate with cognitive function, particularly race, family SES, and neighborhood SES.

Our analysis of the ABCD study revealed that exposure to extreme heat is significantly associated with lower cognitive function. A previous study explored the behavioral health implications of extreme heat exposure, both in terms of individual delinquency and peer delinquency. The study showed that children from Black, low SES families, and those residing in low SES neighborhoods, are more likely to experience heat exposure. Furthermore, exposure to extreme heat was linked to higher peer and child delinquent behaviors. That finding suggested that the most vulnerable groups of children, who are already experiencing considerable amount of economic, environmental, and social disadvantage are the ones at the highest risk of exposure to extreme heat. Thus, extreme heat exposure should be studied in risk-risk and cumulative risk frameworks.

We found that extreme heat exposure is associated with lower cognitive function among US children. This association can be attributed to several factors, including physiological stress responses to heat, which can impair cognitive functioning, self-regulation, and other developmental milestones. Parenting, physical activity, nutrition, and school attendance may be under the influence of heat exposure. Additionally, the discomfort and irritability that extreme heat exposure causes may potentially lead to poor decision-making and impulsivity. Children may reduce their presence and participation in outdoor spaces and activities during heatwaves that may be detrimental to their learning. They may also have different sleep quality that has some effect on cognition. The chronic stress in such conditions may reduce parent – child communication and engagement, or even parental support, which can, in turn, lead to lower cognitive function of children.

### 4.1. Implications

The findings of this study have significant public health and policy implications, particularly in the context of climate change and its growing impact on children’s health and development. As extreme heat events become more frequent and severe, it is crucial to recognize their potential negative effects on cognitive function in children, which can have long-term consequences for educational attainment and socio-emotional development. Given that children from low-income families and marginalized racial groups are disproportionately affected, policies aimed at mitigating the impact of climate change should prioritize vulnerable populations. Interventions could include providing access to cooling infrastructure in schools and communities, expanding green spaces, and promoting public awareness of heat-related risks.

Additionally, the study highlights the need to integrate environmental factors, such as heat exposure, into broader frameworks of childhood development, particularly for populations already experiencing economic and social disadvantages. Addressing these environmental stressors may not only improve cognitive outcomes but also reduce the compounding effects of socioeconomic and racial disparities.

### 4.2. Limitations

This study has several limitations that should be acknowledged. First, the measure of extreme heat exposure was likely generalized and may not account for microclimatic variations in children’s immediate living environments. Factors such as housing conditions, access to air conditioning, and neighborhood infrastructure (e.g., green spaces) were not fully captured, which could influence individual heat exposure and cognitive outcomes.

Second, while the study controlled for a range of socio-demographic factors, other potential confounders, such as access to health care, nutritional status, and parental education, were not extensively explored. These factors could also mediate or moderate the relationship between heat exposure and cognitive function.

Third, the cross-sectional nature of the analysis limits the ability to establish causal pathways. Longitudinal studies would be required to better understand the directionality and duration of the effects of extreme heat on cognitive development.

### 4.3. Future Research

Future research should explore the mechanisms underlying the association between extreme heat exposure and cognitive function. One potential avenue is the role of physiological stress responses, such as elevated cortisol levels, in impairing cognitive processes like attention, memory, and executive function. Investigating how chronic exposure to heat influences neural development, particularly in the prefrontal cortex, could provide more insight into how environmental stressors shape cognitive outcomes over time.

Additionally, future studies should consider the moderating effects of resilience factors, such as social support, access to cooling facilities, and individual coping strategies. Understanding how some children, despite experiencing extreme heat, maintain cognitive function could inform targeted interventions. Moreover, exploring how heat exposure interacts with other environmental and socio-economic stressors—such as air pollution or food insecurity—would enhance our understanding of cumulative risks and protective factors in shaping cognitive development.

## 5. Conclusion

This study contributes to the growing body of literature on the impact of climate-related environmental stressors on child development. By demonstrating that extreme heat exposure is associated with lower cognitive function, particularly in children from vulnerable socio-economic backgrounds, the findings underscore the need for urgent policy action to mitigate the effects of climate change on at-risk populations. Moving forward, it is essential to incorporate environmental factors like heat exposure into broader risk frameworks, particularly when considering the developmental outcomes of children. Through targeted interventions and policy reforms, we can better protect the cognitive and developmental health of children in an era of increasing environmental challenges.

## Figures and Tables

**Figure 1.**
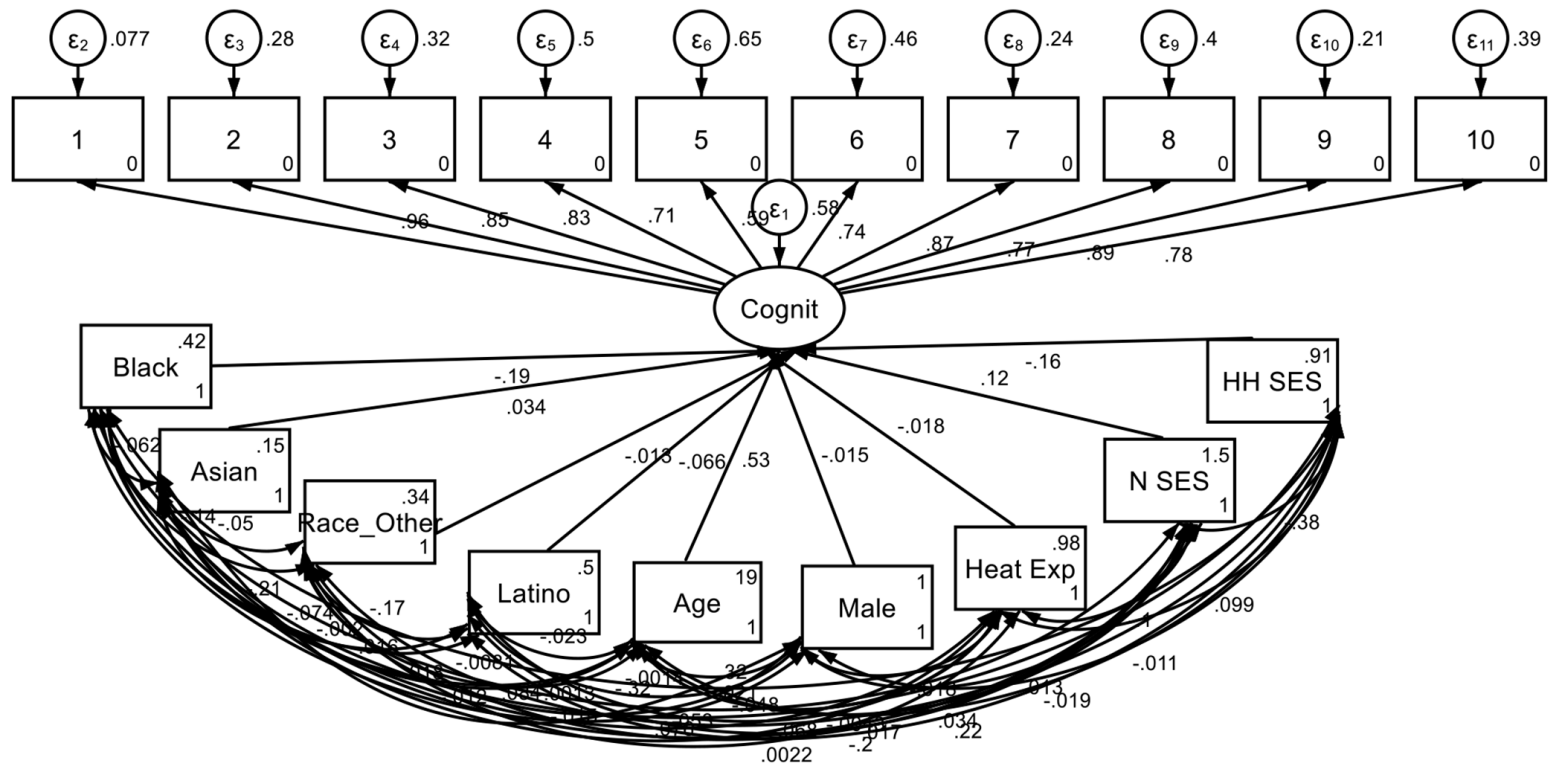
Summary of the Structural Equation Model (SEM) **Note:** 1: Total comp; 2: Reading; 3: Picture vocab; 4: Picture; 5: Pattern; 6: List; 7: Fluid Composition; 8: Flanker; 9: Crystalized; 10: Card Sort)

**Table 1.** Summary of the Structural Equation Model (SEM)

			B	SE	95%	CI	p
Heat Exposure**	→	Cognitive Function	−0.018	0.008	−0.033	−0.003	0.018
Age	→	Cognitive Function	0.525	0.004	0.517	0.533	0.000
Male	→	Cognitive Function	−0.015	0.007	−0.029	−0.001	0.041
Low HH SES	→	Cognitive Function	−0.163	0.009	−0.180	−0.146	0.000
Neighborhood income/50000	→	Cognitive Function	0.121	0.008	0.105	0.137	0.000
Other	→	Cognitive Function	−0.013	0.008	−0.027	0.002	0.099
Latino	→	Cognitive Function	−0.066	0.008	−0.082	−0.050	0.000
Black	→	Cognitive Function	−0.190	0.009	−0.206	−0.173	0.000
Asian	→	Cognitive Function	0.034	0.007	0.019	0.048	0.000
Measurement							
Cognitive Function	→	Total comp	.961	.001	.958	.963	0.000
Cognitive Function	→	Reading	.847	.002	.842	.851	0.000
Cognitive Function	→	Picture vocab	.825	.002	.821	.830	0.000
Cognitive Function	→	Picture	.709	.003	.703	.716	0.000
Cognitive Function	→	Pattern	.595	.004	.588	.602	0.000
Cognitive Function	→	List	.737	.003	.731	.743	0.000
Cognitive Function	→	Fluid Composition	.871	.003	.865	.876	0.000
Cognitive Function	→	Flanker	.772	.003	.767	.778	0.000
Cognitive Function	→	Crystalized	.886	.002	.883	.890	0.000
Cognitive Function	→	Card Sort	.781	.003	.775	.787	0.000

* uncorrected

** reshist_addr1_coi_he_heat
